# Response of *Anopheles gambiae* s.l. (Diptera: Culicidae) to larval habitat age in western Kenya highlands

**DOI:** 10.1186/1756-3305-6-13

**Published:** 2013-01-16

**Authors:** Stephen Munga, John Vulule, Eliningaya J Kweka

**Affiliations:** 1 Centre for Global Health Research, Kenya Medical Research Institute, Nairobi, Kenya; 2 Tropical Pesticides Research Institute, Division of Livestock and Human Diseases Vector Control, Mosquito Section, P.O. Box 3024, Arusha, Tanzania

## Abstract

**Background:**

Larval control is of paramount importance in the reduction of vector populations. Previous observations have suggested that, larvae of *Anopheles gambiae* s.l occur more often in small temporary habitats while other studies showed that long-lasting stable habitats are more productive than unstable habitats. In addition, the physical and biological conditions and stability of larval habitats can change rapidly in natural conditions. Therefore, we examined the effect of larval habitat age on productivity, larval survival and oviposition preference of *Anopheles gambiae*.

**Methods:**

We sampled the three different habitat ages (10, 20 and 30 days) on a daily basis for a period of six months to determine mosquito larval abundance. In addition, we tested the effect of age of water (habitat age) on the oviposition choice preference of *An. gambiae*, larval development time and survivorship, and wing lengths of emerging adults. Additionally, chlorophyll a and abundance of mosquito larval predators in these habitats were monitored.

**Results:**

*Anopheles gambiae* s.l. larvae were significantly more abundant (P=0.0002) in habitats that were cleared every 10 days compared to the other habitats. In particular, there were 1.7 times more larvae in this habitat age compared to the ones that were cleared every 30 days. There were significantly (P<0.001) more mosquito larval predators in the ‘30 day’ habitats compared to the other habitats. Oviposition experiments revealed that significantly more eggs (P<0.05) were laid in fresh water and water that was 5 days old compared to water that was 10 and 15 days old. However, pupation rate, development times and wing lengths of male and female *An. gambiae* in the different habitat ages was statistically insignificant (P>0.05).

**Conclusion:**

The current study confirmed that age of the habitat significantly influences the productivity of malaria vectors in western Kenya highlands. Given that malaria vectors were found in all habitats with varying ages of water, simple environmental methods of maintaining the drainage ditches in the valley bottoms can help reduce larval abundance of malaria vectors. Such inexpensive methods of controlling mosquito breeding could be promoted to supplement other vector control methods, especially in areas where scarce resources are available for intensive mosquito control.

## Background

Larvae and pupae of *Anopheles gambiae* Giles, the principal vector for *Plasmodium falciparum* malaria in sub-Saharan Africa, tends to occur in small, temporary sunlit pools. *An. gambiae* mosquito is an r-strategist and is able to colonize suitable aquatic habitats within a few days after they are created [1]. In small temporary habitats, predators may be less prevalent and larval food may be more abundant than it is in long-lasting habitats [2]. However, other studies have demonstrated that stable habitats are more productive than temporary unstable habitats [3,4]. Further, *An. gambiae* larval habitats usually occur near human habitation, and are often subject to anthropogenic activities [5]. For example, cow footprints, cultivated swamps and drainage ditches form the major breeding habitats for *An. gambiae* in western Kenyan highlands [6,7]. However, little is known about the effect of habitat disturbance or habitat age on mosquito larval development and survivorship.

In the recent years, western Kenya highlands, where this study was conducted, have experienced epidemics of malaria transmission [8]. These highland sites have also experienced dramatic land use changes during the same period [9]. For example, most of the natural swamps have been cultivated for growing agricultural crops, and rain forests have been cleared for crop planting, cattle grazing, commercial logging, firewood and housing construction [10]. The drastic land-use alteration can promote vector-borne disease transmission in several ways [11,12]. First, more human made aquatic habitats become available for the *An. gambiae* complex. Second, the physical and chemical properties of mosquito larval habitats may change. Third, the micro-climatic conditions of mosquito larval habitats may change, favoring rapid development of the malaria vector. Since *An. gambiae* prefers to utilize transient freshwater habitats, thus habitat age may influence mosquito larvae abundance, development and survivorship through its effect on predation and food availability. In this study, we investigated the effects of habitat age on *An. gambiae* s.l. larval abundance and predator abundance. We also characterized the abundance of green algae, which are considered as an important larval food for *An. gambiae*[13-15]. Further, we investigated the influence of habitat age on oviposition preference and development of *An. gambiae* larvae. The information on *An. gambiae* larval population regulation is critical for the development of rational and cost-effective larval control methods.

## Methods

### Study site

The study site is located in Iguhu village (34°45^′^ E, 0°10^′^ N), Kakamega district, a highland area in western Kenya. The altitude of the study site ranges from 1,420 to 1,580 m above sea level and the Yala River transects the study site. The area is characterized by undulating topography, with both steep and gently sloping hills. Although most of the study area has been cultivated, small patches of indigenous forest still remain along the rivers and streams. The hill is mostly dotted with maize plots and patches of tea plantation, while several wetlands are located along the Yala River valley. The average annual rainfall is about 1,950 mm, while peak rainfall generally occurs between March and June followed by a short rainy season in October and November.

### Effects of habitat age on *An. gambiae* s.l. abundance

We determined the effects of habitat age on mosquito larval abundance in western Kenya highlands for a period of six months from September 2005 to February 2006. Thirty (30) larval habitats in a low lying area near a stream at Iguhu and each habitat measured 7 × 0.5 m in length and width respectively. The three treatments had a total of ten replicates each: (1) habitats were cleared of grass and water was replenished from the local stream every 10 days (frequent disturbance); (2) habitats were cleared of grass and water was replenished from the local stream every 20 days (intermediate disturbance); and (3) habitats were not disturbed during the 30 day period (no disturbance). This experiment was conducted in one month (30 days) after which the no-disturbance habitats were also cleared of water and grass and the experiment repeated again. The 30 larval habitats were randomly assigned the treatments (frequent, intermediate and no disturbance) at the beginning of the experiments and kept the same for the study period. Larval mosquito abundance in the habitats was determined daily by making 50 dips per habitat using 350 ml standard dippers. Larvae of *An. gambiae* s.l. occurring in each habitat were counted and scored into instars [1,15]. Further, we characterized habitat size (length, width, and depth).

### Effects of habitat age on larval development

In addition, we determined the effect of larval habitat age on larval development of *An. gambiae* s.l. using artificial habitats created in plastic washbasins (35 cm in diameter and 15 cm deep). The artificial habitats were created by mixing 2 kilograms of dry soil and 2.5 liters of water in each washbasin. The dry soil was collected from a farmland within the study area. Two holes (3 cm in diameter) were drilled 1 cm from the top edge of the washbasin and covered with a screen (mesh size = 200 μ) to maintain a constant water level during periods of rain while preventing larvae from being washed away. On the first day of the experiment, 6 replicates were created and covered with mosquito netting to prevent wild mosquitoes from laying eggs in the experimental washbasins. These washbasins were left in the open area to mimic the natural habitats.

Six other treatments were created on day 5, 10, or 15 and treated the same way as artificial habitats above. The experiment was conducted from January to February 2006. A cohort of 20 first-instar *An. gambiae* s.s. larvae (1–3 hrs old) from a laboratory-reared colony that originated from the study area were introduced to these habitats. The habitats were covered with mosquito netting materials to prevent egg laying by other mosquitoes, and randomly placed in natural habitats in an open area within the study site. On a daily basis, we monitored the number of mosquito larvae and pupae in these artificial habitats and the larvae were scored into developmental stages. Water lost through evaporation from the habitats was replaced by adding water of the same age kept in same condition as that used in the experiment. Once the larvae started pupating, the washbasins were examined twice daily from 09 00 hr to 12 00 hrs and from 15 00 hrs to 19 00 hrs for the presence of pupae, and pupae were removed and held in paper cups to allow them to emerge into adults. The emerged adults were then separated by sex and wing lengths measured to the nearest 0.01 mm with an ocular micrometer from the distal end of the alula to the wing tip excluding the fringe scales [16].

### Habitat age and oviposition site selection of *An. gambiae* s.s

We used 4 oviposition substrates in this experiment: water of the following ages 0 days (fresh rainwater, 5, 10 and 15 days old. One hundred ml substrate water was added to a 300 ml transparent plastic cup (the mouth of the cup was 6.4 cm in diameter). A piece of white filter paper was placed tightly in each cup by folding the paper edge upward. This way, all eggs were laid onto the filter paper making it easy to count. The four cups were placed randomly at the corners of the cage. Twenty *An. gambiae* s.s. mosquitoes, 36 hr post blood-feeding, were introduced to the oviposition cage (30 × 30 × 30 cm in size) that contained the four substrates, and 2% sugar solution was provided daily. The adult mosquitoes used in this experiment were from a laboratory-reared colony that originated from the study area. The experiment was set up at 18 00 h, and eggs deposited in each oviposition substrate were counted under the microscope at 10 00 h on the following day. This experiment was conducted in a room at the study site and thus there were no controls for lighting, humidity and temperature. The experiment was repeated 5 times, with 10 replicates per trial.

### Statistical analysis

We used Poisson regression with repeated measures to test for the differences in the number of immature stages of *An. gambiae* s.l. among the three habitat ages. We also tested if the differences in the predator density between the habitat ages were statistically significant, using the analysis of variance with repeated measures. When the effects were significant, Tukey-Kramer multiple comparison tests were used to compare mean values among the habitats. One way analysis of variance (ANOVA) was used to test whether the differences between pupation rate, development time and wing length of resulting *An. gambiae* among water treatments were statistically significant. Pupation rate was defined as the proportion of first-instar larvae that developed to the pupal stage. Development time is defined as the length of time that a first-instar larva requires to emerge as an adult. Finally, we used one-way ANOVA to test if there were any significant differences in the number of eggs that were oviposited in the different ages of water. The Tukey-Kramer multiple comparison test was used to determine the treatments that were significantly different from each other.

## Results

### Effects of habitat age on larval abundance

The species composition and larval abundance of mosquitoes sampled during the study period are presented in Table 1. A total of 50,896 mosquito immature stages were sampled over the study period. Of these, *An. gambiae* s.l. comprised 74.5%, *An. funestus* 3.9%, *An. coustani* 0.5% and *An. implexus* 0.5% and the rest were culicines. The mean number of larval and pupal stage of *An. gambiae* s.l. mosquitoes was significantly influenced by habitat age (χ^2^ = 17.05, df = 2, *P* = 0.0002; Table 1). Comparison between the 10 and 30 day treatments revealed a significant 1.7 fold increase (P<0.001) in immature *An. gambiae* in the 10 day treatment (Table 1). Similarly, habitats cleared after 20 days were 1.3 times more productive (P<0.05) than the non-disturbed habitats (Table 1).

**Table 1:** Abundance of immature stages of anopheline mosquito species and unidentified culicines collected during the study period under the different habitat ages

**Species**	**10 day**	**20 day**	**30 day**
*Anopheles gambiae* s.l.	15589^a^	12670^b^	9637^c^
*Anopheles funestus* s.l.	351^a^	524^a^	1105^b^
*Anopheles coustani*	47	30	195
*Anopheles implexus*	70	85	104
Culicines	3185	3525	3779
Total	19242	16834	14820

*Values with different letters in a row were statistically significant different from each other.

The number of predators occurring in a habitat significantly influenced the number of larvae present (χ^2^ = 59.37, df = 3, *P*<0.0001). Habitats that had fewer predators were 2 times more productive compared to habitats with more predators. Predator density in the 30 day habitats was significantly higher (F _2, 181_ = 8.43, *P*<0.001) compared to the other habitat ages. Tukey HSD test revealed that there were more predators in 30 day habitats than in the 10 day (1.10 vs. 0.14 predators per dip) and 20 day habitats (1.10 vs. 0.64 predators per dip) (Table 2). Chlorophyll a level in water significantly influenced (χ^2^ = 59.37, df = 3, *P*<0.0001) larval and pupal abundance. In general, habitats that had a higher chlorophyll a value were more productive compared to habitats with low values. Tukey HSD test revealed that there were significant differences in the levels of chlorophyll a among the habitats (Table 2).

**Table 2:** Larval, predator and chlorophyll a abundance (mean ± SE) in different water habitats

**Habitat age**	**Number of larvae per dip**	**Number of predators per dip**	**Chlorophyll a (ppm)**
10 day	0.191 ± 0.006^a^	0.14 ± 0.06^a^	73.3 ± 1.16^a^
20 days	0.155 ± 0.005^b^	0.64 ± 0.17^b^	67.4 ± 1.06^b^
30 days	0.118 ± 0.004^c^	1.10 ± 0.32^c^	87.1 ± 1.79^c^

* Values above are the mean values (± SE) and the letters following the numerical values indicate the results of multiple comparison tests. The values with different letters in a column were significantly different from each other.

### Effects of habitat age on larval survivorship and oviposition site selection of *An. gambiae* s.s

**Figure 1:**
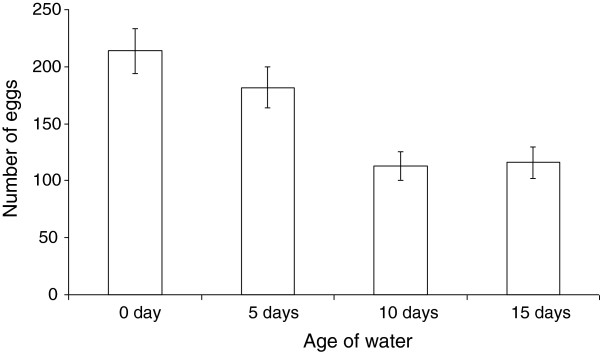
The number of eggs (mean ± SE) oviposited in water from the four different habitat age types.

Pupation rate did not vary significantly among the water of the different ages (F _3, 23_ = 0.62, P = 0.42). The highest mean pupation rate (61%) occurred in freshwater (day 0) while the lowest pupation rate (50%) occurred in water of day 5 (Table 3). Female and male development times and wing length measurements were statistically insignificant (*P*>0.05) among habitat ages (Table 3). However, age of water significantly influenced the number of eggs laid (F _3, 199_ = 9.42, *P*<0.001). In particular, significantly more eggs were laid in freshwater and 5 day old water (mean= 213.9 and 181.5 respectively) compared to 10 and 15 day water (mean= 112.7 and 115.8 respectively) (Figure 1). The number of eggs laid in water of day 0 and 5 did not differ statistically. Similarly, the number of eggs laid in water that was 10 and 15 days old were statistically insignificant (P>0.05).

**Table 3:** Mean ± SE pupation rates, development times and wing lengths from the larval survival experiment

**Variable**	**Day 0**	**Day 5**	**Day 10**	**Day 15**
Pupation rate	0.61 ± 0.06	0.50 ± 0.06	0.53 ± 0.06	0.55 ± 0.06
Male development time (days)	10.21 ± 0.19	10.59 ± 0.20	10.71 ± 0.22	10.90 ± 0.24
Female development time (days)	10.75 ± 0.24	11.01 ± 0.23	10.39 ± 0.22	10.81 ± 01.6
Female wing length (mm)	2.80 ± 0.02	2.83 ± 0.02	2.83 ± 0.02	2.80 ± 0.04
Male wing length (mm)	2.70 ± 0.02	2.75 ± 0.02	2.76 ± 0.02	2.70 ± 0.02

All comparisons were statistically insignificant.

## Discussion

This study demonstrated that habitat age significantly influenced the abundance of *An. gambiae* s.l. larvae occurring in water of different ages. We found significantly higher numbers of mosquito larvae in water replenished frequently compared to the water replenished after 30 days. Several factors may regulate the population dynamics of mosquito larvae in a habitat. One such important factor is the predation effect in relatively stable habitats [17-19]. We found abundant predators such as coleopterans and hemipterans in all the habitats, with significantly higher predator abundance in habitats with older water (replenished after 30 days). It has been suggested that larval mortality is lower in smaller temporary habitats than it is in large, permanent habitats where the predation rate is high [2], thus the effect of predation may have significantly reduced the number of mosquito larvae in these older habitats. Although, predatory effect may be higher in such habitats, productivity may be regulated by other factors such as the availability of algae, as demonstrated by this study. Thus older habitats may still provide suitable breeding habitats for immature stages of malaria vector. For example, in urban Malindi town at the Kenyan coast, unused swimming pools were found to play a significant role in the breeding of *Anopheles* immature stages [20]. Other studies in western Kenya highlands have demonstrated that, drainage ditches had significantly higher productivity in all seasons compared to other habitat types [6,7], thus productivity of malaria vectors from drainage ditches can play a role in increasing the risk of malaria transmission. Greater abundance of mosquito larvae in habitats with higher chlorophyll a values also confirmed the importance of chlorophyll a in the diet of larvae. Previous studies have demonstrated the importance of chlorophyll a as a dietary content of *An. gambiae* larvae [13,14]; consequently food resources may be a limiting factor to production of adult mosquitoes. More stable habitats may have abundant food resources, however, habitat productivity may be influenced by other factors rather food resources. For example, we found high levels of chlorophyll a in habitats that were older compared to 10 and 20 days old. Though the levels of chlorophyll a were higher in 30 day old habitats, larval abundance was significantly lower compared to 10 and 20 day old habitats. This can be attributed to higher predation pressure since we found higher predator density in 30 day habitats.

The results from the present oviposition experiments demonstrated that water conditions additionally affected oviposition preference of *An. gambiae* s.l. In particular, *An. gambiae* mosquitoes deposited significantly more eggs in freshwater (day 0) compared to other ages of water. We also observed that the number of eggs deposited in water of day 0 and 5 days were statistically insignificant. Immature stages of this mosquito occur in small, sunlit pools such as borrow pits, hoof prints, tire tracks, drainage ditches, and small puddles [1,4,19,21], which may be very temporary in nature. We have also previously demonstrated that *An. gambiae* prefers to lay eggs in small pools filled with rainwater [22]. It is interesting to note that differences in pupation rate, male and female development time and male and female wing length were statistically insignificant among the different ages of water. This difference could be due to experimental design, as each treatment only differed in age by a few days. Thus, it is possible that the time intervals used may have been too short to detect a difference.

Taken together, our findings suggest that simple environmental methods such as keeping the water in the drainage ditches moving could reduce the productivity of malaria vectors in drainage ditches in valley bottoms of western Kenya highlands. A recent study at the same site demonstrated that cropping cycle (land preparation, crop weeding and plant flowering) influenced larval abundance [23]. As the cropping cycle progresses at this study site, the farmers have a practice of not clearing the drainage ditches in their farms and small pools of stagnant water become suitable breeding habitats for malaria vectors. However, if these habitats are cleared quite often and water is left to flow all the times, this would make it less suitable for breeding of these vectors. Since mosquito larvae were found in habitats of all ages, this may suggest that making the water in these drainage ditches to flow is likely to reduce the abundance of mosquito larvae. Other studies have demonstrated that the likelihood of finding *Anopheles* mosquitoes is higher in drains compared to water flowing at normal velocity [24]. Although *An. gambiae* prefers to breed in clear sunlit pools of water, ensuring that the water in the drainage ditches is flowing would reduce oviposition in those habitats. Similarly, moving water is likely to have little amount of algae, which is food to the mosquito larvae, thus these habitats would be less suitable for breeding. Older habitats were also found to have malaria vectors. These habitats are likely to be organically polluted; however *Anopheles* species have been demonstrated to breed even in highly organically polluted habitats [25]. Thus all habitats need to be considered as habitats for malaria vectors in any control program. A previous study in this area demonstrated that predation is one of the most important factors regulating the productivity of malaria vectors from relatively stable habitats [6]. The importance of predation has also been illustrated by other studies [2,18,19]. Additionally, the reduction in eggs oviposited in older habits resulted in a lower productivity from such habitats.

## Conclusion

Since malaria vectors were found in all habitats with varying ages of water, these results suggest that simple environmental methods of maintaining the drainage ditches in the valley bottoms can help reduce productivity of these vectors. Such inexpensive and simple methods to control the breeding of malaria vectors could be promoted to supplement other vector control methods, especially in areas where resources for vector control are scarce and habitats are aggregated in the valley bottoms.

## Competing interests

The authors declare that they have no competing interests.

## Authors’ contributions

SM conceptualized and designed the study, collected the data and drafted the manuscript. JV, EJK were involved in the conception of the study and the drafting of the manuscript. All authors read and approved the final version of the manuscript.
